# RNA Preservation Agents and Nucleic Acid Extraction Method Bias Perceived Bacterial Community Composition

**DOI:** 10.1371/journal.pone.0121659

**Published:** 2015-03-23

**Authors:** Ann McCarthy, Edna Chiang, Marian L. Schmidt, Vincent J. Denef

**Affiliations:** Department of Ecology and Evolutionary Biology, University of Michigan, Ann Arbor, MI, United States of America; University of Illinois at Chicago, UNITED STATES

## Abstract

Bias is a pervasive problem when characterizing microbial communities. An important source is the difference in lysis efficiencies of different populations, which vary depending on the extraction protocol used. To avoid such biases impacting comparisons between gene and transcript abundances in the environment, the use of one protocol that simultaneously extracts both types of nucleic acids from microbial community samples has gained popularity. However, knowledge regarding tradeoffs to combined nucleic acid extraction protocols is limited, particularly regarding yield and biases in the observed community composition. Here, we evaluated a commercially available protocol for simultaneous extraction of DNA and RNA, which we adapted for freshwater microbial community samples that were collected on filters. DNA and RNA yields were comparable to other commonly used, but independent DNA and RNA extraction protocols. RNA protection agents benefited RNA quality, but decreased DNA yields significantly. Choice of extraction protocol influenced the perceived bacterial community composition, with strong method-dependent biases observed for specific phyla such as the Verrucomicrobia. The combined DNA/RNA extraction protocol detected significantly higher levels of Verrucomicrobia than the other protocols, and those higher numbers were confirmed by microscopic analysis. Use of RNA protection agents as well as independent sequencing runs caused a significant shift in community composition as well, albeit smaller than the shift caused by using different extraction protocols. Despite methodological biases, sample origin was the strongest determinant of community composition. However, when the abundance of specific phylogenetic groups is of interest, researchers need to be aware of the biases their methods introduce. This is particularly relevant if different methods are used for DNA and RNA extraction, in addition to using RNA protection agents only for RNA samples.

## Introduction

Molecular analyses performed directly on nucleic acid extracts from environmental samples eliminate the biases associated with culture-dependent approaches [1], but introduce a variety of other biases that create differences between real and perceived community composition [2,3]. Because biological variability tends to be higher than technical variability, this does not preclude the relative comparisons between samples on which most studies focus [4]. However, when more subtle differences are of interest, technical biases can confound biological interpretations [5].

Previous studies have shown differences in observed community composition due to sample storage conditions [6], extraction method [7–13], sequencing protocol and user bias [5,14–17], and sequence analysis approach [5,18,19]. Another potential source of bias is differential treatment of DNA and RNA extracts. A majority of studies comparing DNA and RNA (cDNA) sequencing data use different extraction protocols to acquire the DNA and RNA fractions [20–25] instead of avoiding extraction differences by using a combined extraction protocol for DNA and RNA [26–28]. Therefore, it is important to understand how the use of different extraction methods biases overall community composition as well as DNA and RNA levels for specific taxa. In addition to extraction method biases, it has also been argued that the heterogeneity of natural environments necessitates the extraction of all biomolecules from the same sample, using one lysis method to break open the cells prior to separating DNA, RNA, and other molecules of interest [29].

This study aimed to optimize and compare a combined protocol for DNA and RNA extraction (extendable to protein and metabolites [28]) to other commonly used DNA and RNA extraction protocols for aquatic samples. As samples taken for DNA extraction tend to be stored differently than those taken for RNA extraction, we evaluated the effect of different preservation methods on yield, RNA quality, and bacterial community composition. Finally, we determined the relative abundance of bacteria from the phylum Verrucomicrobia using catalyzed reporter deposition fluorescence *in situ* hybridization (CARD-FISH) to validate the high relative abundance of this phylum detected by 16S rRNA gene sequencing data of DNA obtained using our optimized combined DNA/RNA extraction protocol. We selected Verrucomicrobia, as bacteria from this phylum have been differentially represented in sequencing data depending on the extraction protocol used, and because its predominance and potential importance in carbon cycling in both soil and aquatic systems has long been overlooked [30,31].

## Results and Discussion

We used samples from three different freshwater systems for comparing extraction and preservation methods and from a fourth freshwater system for comparing the optimized, combined DNA and RNA AllPrep extraction method to CARD-FISH data (Table 1). To determine yield, quality, and community composition for each protocol, we performed triplicate extractions of segments of the same 142 mm 0.22 μm filter used to collect 3 μm pre-filtered water. One sequencing library was generated from each of these triplicate extracts and submitted for MiSeq 2x250bp sequencing of the V4 region of the 16S rRNA gene.

**Table 1:** Overview of performed experiments.

	**Douglas Lake**			**Lake Huron**			**Huron River**		
	*Yield*		*16S*	*Yield*		*16S*	*Yield*		*16S*
	*DNA*	*RNA*	*Sample name*	*DNA*	*RNA*	*Sample name*	*DNA*	*RNA*	*Sample name*
**Choice of DNA/RNA extraction protocols**									
*Mirvana protocol (RNA/miRNA)*	n/a	0.65	N/A						
*Enzymatic protocol (DNA)*	0.79	n/a	DL-Enz (1)						
*Bead-beating-based protocol (DNA)*	2.21	n/a	DL-Bead (1)						
*AllPrep standard protocol (DNA/RNA/miRNA)*	1.01	0.86	DL-APS (1)						
**Optimization of AP protocol**									
Control 1: AllPrep standard protocol with lysozyme	1.13	0.68	N/A						
* Control 1 without QIAshredder*	0.83	0.32	DL-APS-NQ (1)						
* Control 1 with bead-beating (TissueLyser)*	0.57	1.14	DL-APS-TL (1)						
* Control 1 with beads during vortexing*	0.69	0.98	N/A						
Control 2: RNAlater preserved, AllPrep standard protocol with 1/12th filter, not cut				0.04	0.63	LH-APO-RL (2)	1.07	1.11	N/A
* Control 2 with 2X DNA loading*							0.91	1.00	N/A
* Control 2 with 2X DNA/RNA elution*							1.70	1.28	N/A
* Control 2 with lysozyme*				0.06	0.39	LH-APO-RL-LYS (2)			
* Control 2 with 1/6 of 142 mm filter*				0.03	0.68	N/A			
AllPrep optimized protocol	0.99	1.65	DL-APO-RL (2)						
* AllPrep optimized protocol without lysozyme*	0.87	1.55	N/A						
**RNA preservation method**									
Control 3: no preservative, AllPrep optimized protocol				n/a		LH-APO-NT (2)	0.84	1.08	HR-APO-NT (3)
* Control 3 with RNAlater preservation*				n/a		LH-APO-RL (2)	0.29	0.90	HR-APO-RL (3)
* Control 3 with RNAprotect preservation*							0.20	1.05	HR-APO-RP (3)
* Control 3 with RLT+ lysis buffer preservation*							0.47	0.94	HR-APO-BU (3)
**Sequencing Run**									
*Enzymatic protocol*	0.79	n/a	DL-Enz (1,2,3)						

**Fig 1:**
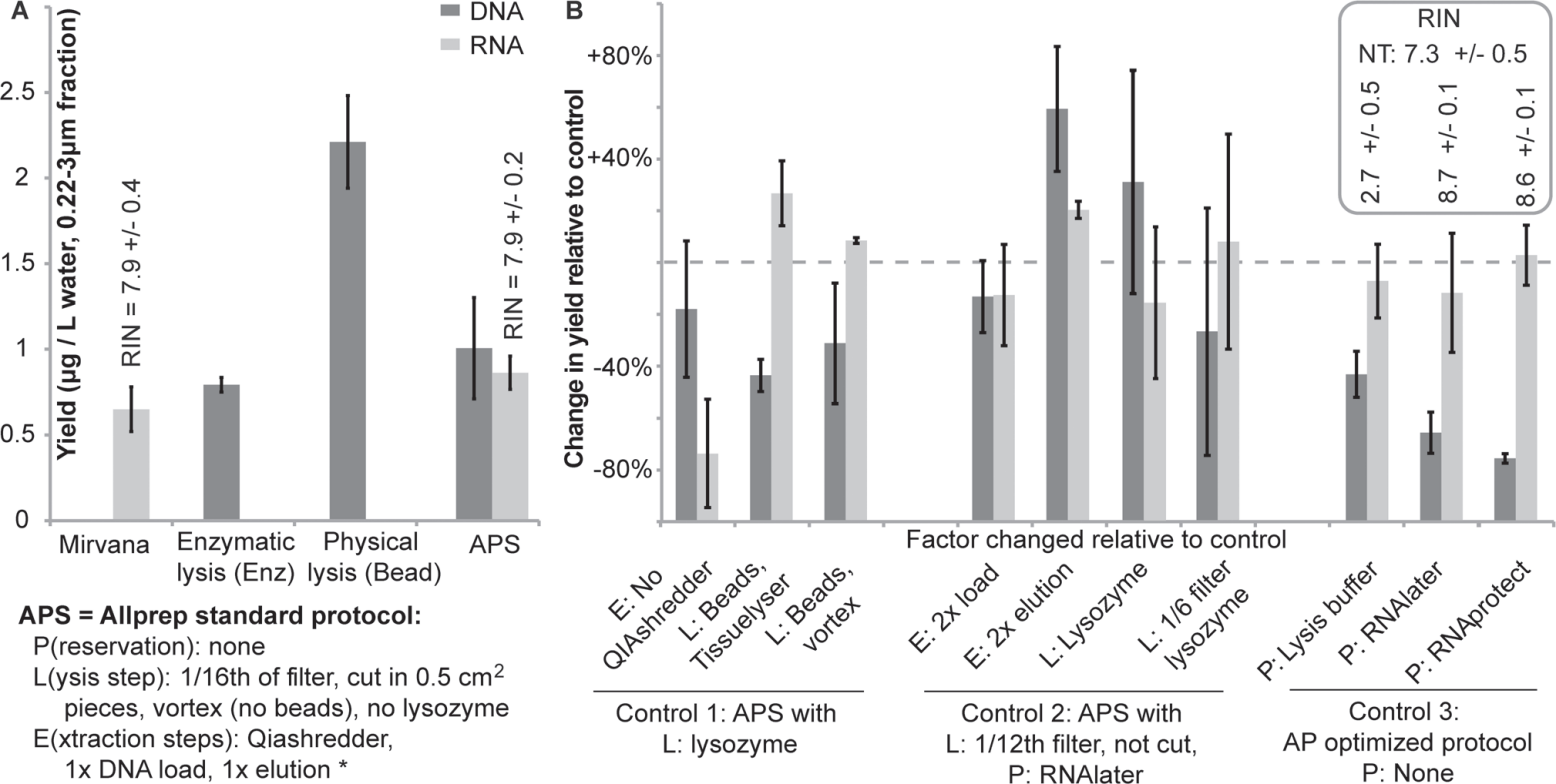
Comparison of DNA/RNA yields and RNA quality (inset). (A) Comparison between the AllPrep standard protocol (APS; described below figure), enzymatic lysis (Enz), enzymatic/bead beating (Bead), and Mirvana extraction protocols (Mirvana). (B) Comparison between different iterations of the AP protocol, examining the effect of preservation method {P}, lysis conditions {L}, and other extraction modification steps {E}. The data presents changes in yield relative to a control, where each column shows the effect of one factor relative to a control that differed in that factor only. The control was extracted using either the AP standard protocol or a modification as indicated by the X-axis labels below the horizontal lines. The factor that was changed is indicated just below the data column. The dotted line indicates no change relative to control. All data represent averages of three extractions, with error bars indicating the 95% confidence interval, except for the effect of lysozyme, which averaged the relative yield effects from multiple samples (from DL, LH, and HR samples, three replicate extractions each). Inset: RIN = RNA integrity number quality (0 (poor)- 10 (high) range) assessment by Bioanalyzer; NT = no preservation treatment.

The protocols that were compared and the samples that were used for each of the comparisons are listed. Yields are the average amount of DNA or RNA (μg) extracted per liter of filtered water (n/a when protocol did not extract DNA or RNA). For lysozyme treatment, the relative yield comparison in Fig. 1B was based on sample comparisons from Douglas Lake (AP optimized with and without lysozyme) and Lake Huron (Control 2 experiments). No yield data was included for the comparison of LH-APO-NT to LH-APO-RL as these derived from separate filters on which different water volumes were filtered. The abbreviations in the 16S columns correspond to those used in the figures: the first 2 letters indicate the sample site, the next 3–4 the extraction method used, and the remaining letters indicate optimizations to the protocol or differences in preservation procedure. The number(s) in parentheses indicates the sequencing run that data were generated on. N/A indicates samples were extracted for yield evaluation but not submitted for sequencing. Not included in the table are samples from Muskegon Lake that were used to compare CARD-FISH and sequencing data derived from RNAlater preserved, AllPrep optimized protocol extracted samples (ML-APO-RL (4)).

### Extraction yields

We compared the combined Qiagen AllPrep DNA/RNA/miRNA protocol (AP), which has been used for extraction of aquatic microbial community samples [28,32,33], to two DNA-only protocols (enzymatic (Enz) and bead-beating (Bead)) and one RNA-only protocol (Mirvana) that have been commonly used for the extraction of nucleic acids from aquatic samples [25,34,35]. DNA and RNA yields of the AP combined protocol compared favorably to the other protocols (Fig. 1A; Table 1). Only the Bead DNA extraction protocol, which relies on harsh bead beating, resulted in a higher yield. The RNA quality of the yield from the combined and separate protocols did not differ. All extraction protocols generated DNA of similar integrity (band on agarose gel > 10kb), though the AP (9,225 ± 2,851 (95% confidence interval) sequences) and Enz protocols (6,535 ± 2,195 sequences) generated significantly more sequencing reads than the Bead protocol (1,135 ± 66 sequences) despite library normalization after PCR using the SequalPrep^TM^ kit. A possible explanation is that partial inhibition during PCR on the three replicate extracts using the Bead protocol resulted in DNA yields below the maximum binding capacity of the SequalPrep kit, resulting in lower representation of these libraries in the sequencing run.

We modified the AP combined protocol in an attempt to increase yields by adding the beads used in the Bead DNA protocol, using either vortexing or the AP manufacturer’s recommended bead beater (TissueLyser). These modifications increased the RNA yield, but decreased the DNA yield (Fig. 1B). Use of the QIAshredder column, which is intended to increase RNA yields, is essential as its omission reduced RNA yields while maintaining DNA yield.

We tested the effect of RNA protection agents on RNA quality and yield for the AP protocol. In addition to a no preservative control we also tested preservation of the filter in lysis buffer + β-mercaptoethanol. The use of RNAlater or RNAprotect improved RNA quality, but did not affect RNA yield (Fig. 1B). However, all filters treated with a preservative yielded significantly less DNA. A previous study, using extraction methods different from ours, reported similar reductions in DNA yields, but also found reduced RNA yields due to RNAlater storage [36]. To test whether loss of DNA yield was due to inhibition of binding to the silica column caused by the preservation agents (the DNA column is the first column used in the protocol), we tested the effect of reloading the lysate onto the silica column twice. Similarly, we tested whether RNA preservatives inhibited DNA release from the column by eluting the bound DNA twice. Double elution increased the DNA yield by 60% and RNA yield by 20%, while double loading did not significantly change DNA or RNA yield. Finally, though results were highly variable from sample to sample, we showed that lysozyme treatment and increasing the fraction of the 142 mm filter used in the extraction did not significantly change the DNA and RNA yield (Fig. 1B). The variability we observed between different samples is consistent with previous reports indicating reduced DNA yields when using lysozyme [37].

The optimized AP protocol used intact rather than 0.5 cm^2^-fragmented filters, a short wash step in PBS to remove excess RNAlater, a five minute lysozyme incubation, and two DNA and RNA elution steps (see details in methods section). DNA yields from the 0.22–3 μm fraction using the this optimized protocol ranged from 95 ± 8 ng DNA L^-1^ in oligotrophic bottom water of Lake Michigan (110 m depth) to 1,871 ± 410 ng DNA L^-1^ in mesotrophic Huron River water. For RNA, yields ranged between 94 ± 1 ng RNA L^-1^ from oligotrophic bottom water of Lake Michigan (110 m depth) to 1,892 ± 80 ng RNA L^-1^ from a mesotrophic freshwater estuary (Muskegon Lake, MI). Yields are highly dependent on microbial biomass at the time of sampling, and while our yields are well below the highest extraction yields that have been reported [37], they are within the range of many reported yields from marine and freshwater samples using a variety of methods [28,38,39].

### Community composition

**Fig 2:**
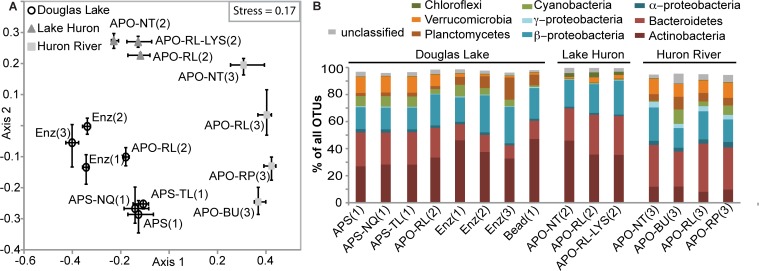
Analysis of technical bias to perceived community composition. (A) NMDS of the 16S rRNA gene amplicon sequencing data based on a Bray-Curtis dissimilarity matrix generated after random subsampling of 2,800 sequences from each sample. Bars indicate the range of coordinates for the three replicate extractions/sequencing datasets per treatment. (B) Phylum level data (top 10 most abundant phyla, fractions of all reads). Number 1–3 between parentheses indicates the sequencing run from which each dataset is derived. APO combines three slightly different treatments that did not result in significantly different taxonomic representations (S2 Fig.). Acronyms: Extraction protocols: APS (standard AllPrep protocol), APO (optimized AllPrep protocol), Enz (Enzymatic protocol), Bead (Bead-beating protocol); Preservation methods: NT (none), RL (RNAlater), RP (RNAprotect), BU (Qiagen Lysis Buffer RLT+); Other modifications: LYS (lysozyme), NQ (No QIAshredder column), TL (bead-beating with TissueLyser). Except for the APO samples, none of the Douglas Lake filters were preserved in RNA protection agents.

A non-metric multidimensional scaling (NMDS) ordination of the 16S rRNA gene amplicon sequence data indicated that the primary grouping of the samples was based on sample origin (Fig. 2A). The average Bray-Curtis dissimilarity—corrected for within-treatment (technical replicates) dissimilarity—between communities observed in samples originating from different sites was 0.33 (± 0.06, 95% confidence interval). Dissimilarity between communities observed in samples originating from the same site, but extracted using different methods was 0.17 (± 0.05), stored using different preservation procedures was 0.08 (± 0.02), and analyzed on independent sequencing runs was 0.08 (± 0.05)(Table 2). When grouping the data by sampling site while ignoring technical differences, AMOVA analysis indicated the communities to be significantly different from each other (p < 0.0001, Table 2). Thus, technical biases were smaller than the biological differences between communities in this study.

**Table 2:** Significance analysis of community composition differences.

**Treatment 1**	**Treatment 2**	**p-value**	**within (1)**	**within (2)**	**between**
*Different sampling sites*					
DL-AP-ALL	HR-AP-ALL	**<0.0001**	0.18	0.27	0.64
DL-AP-ALL	LH-AP-ALL	**<0.0001**	0.18	0.14	0.46
DL-Enz-ALL	HR-AP-ALL	**<0.0001**	0.16	0.27	0.58
DL-Enz-ALL	LH-AP-ALL	**<0.0001**	0.16	0.14	0.37
HR-AP-ALL	LH-AP-ALL	**<0.0001**	0.27	0.14	0.62
DL-ALL	HR-AP-ALL	**<0.0001**	0.17	0.27	0.59
DL-ALL	LH-AP-ALL	**<0.0001**	0.17	0.14	0.40
*Different extraction methods*					
DL-AP-ALL	DL-Enz-ALL	**<0.0001**	0.18	0.16	0.35
DL-APS	DL-APO-RL	**0.0029**	0.19	0.14	0.26
DL-APS	DL-Enz-ALL	**<0.0001**	0.19	0.16	0.39
DL-APO-RL	DL-Enz-ALL	**0.0039**	0.14	0.16	0.32
LH-APO-RL	LH-APO-RL-LYS	0.1918	0.13	0.16	0.15
*Different preservation methods*					
LH-APO-NT	LH-APO-RL	0.1035	0.13	0.13	0.19
LH-APO-NT	LH-APO-RL-LYS	0.0781	0.13	0.16	0.21
LH-APO-NT	LH-APO-RL/RL-LYS	**0.0057**	0.13	0.14	0.20
HR-APO-NT	HR-APO-BU	**0.0098**	0.28	0.27	0.39
HR-APO-NT	HR-APO-RL	**0.0116**	0.28	0.26	0.31
HR-APO-NT	HR-APO-RP	**0.0122**	0.28	0.29	0.37
HR-APO-BU	HR-APO-RL	0.1009	0.27	0.26	0.37
HR-APO-BU	HR-APO-RP	0.1055	0.27	0.29	0.35
HR-APO-RL	HR-APO-RP	0.1027	0.26	0.29	0.35
*Different sequencing runs*					
DL-Enz(1)	DL-Enz(2)	**0.0167**	0.16	0.14	0.23
DL-Enz(1)	DL-Enz(3)	0.0992	0.16	0.17	0.29
DL-Enz(2)	DL-Enz(3)	0.1028	0.14	0.17	0.19

Results of AMOVA analysis based on 10,000 iterations. Acronyms as in Table 1 except for ALL (all samples extracted with particular extraction protocol or from particular sample site). The table presents the average community dissimilarity (Bray-Curtis dissimilarity) between replicates (within treatment 1 and within treatment 2) and between different treatments.

There was a significant effect of extraction method, with the Enz, standard AP, and optimized AP protocol all resulting in significantly different observed community structures (AMOVA, p < 0.05). We had to reduce the subsampling level to include the Bead protocol data (S1 Fig.). Lower subsampling did not significantly change the ordination (S1 Fig.), which is in line with previous analyses [40], and the community structure as revealed through the Bead protocol was significantly different from the AP protocols, but not from the Enz protocol (AMOVA, p < 0.05). Addition of lysozyme or bead beating during the lysis step of the AP protocol did not significantly change the perceived community structure (Table 2, Fig. 2, S2 Fig.). Although community analyses of cDNA are not presented in this study, we have been able to successfully generate 16S rRNA gene amplicon sequence data from DNA and RNA from Lake Michigan communities using the AP protocol. In a recent sequencing effort, amplicon sequence data were generated from 83/92 DNA and 91/92 cDNA libraries, generating an average of 272,502 (± 3,637 standard error) and 95,133 (± 1,729) read pairs per sample, respectively.

All methods revealed dominance by the three groups typically dominating freshwater samples (Actinobacteria, β-Proteobacteria, Bacteroidetes [31]: Fig. 2B). Changes in perceived community composition due to extraction protocol differences were attributed to lower observed levels of Actinobacteria, β-Proteobacteria, Planctomycetes, and higher Bacteroidetes and Verrucomicrobia levels for the AP protocol compared to the Bead and Enz protocol. Specific OTUs identified by Metastats as driving differences in overall community composition mostly belonged to these 5 phyla (S1 Table). The technical biases affecting the relative abundance of specific phylogenetic groups most likely originates from differences between the lysis conditions of the three extraction methods, as has been suggested in previous studies [10,41].

The striking differences between the relative abundance of Verrucomicrobia and Planctomycetes in the sequencing libraries originating from different extraction protocols are consistent with reports on their prevalence in freshwater systems [31]. Fluorescence *in situ* hybridization (FISH)-based studies have shown that Verrucomicrobia can be abundant in lakes [42], and our work indicates that the range of observed relative abundances of bacteria from this phylum is in part due to marked differences in phylum-level extraction yields from different extraction protocols. Tsementzi et al. [41] recently demonstrated that cDNA levels of Verrucomicrobia were underrepresented relative to DNA levels in studies of freshwater ecosystems. In that study, independent extraction methods were used to recover DNA and RNA, and extraction biases may partially explain this observation [41]. To explore this discrepancy more closely, we compared Verrucomicrobia levels detected in amplicon surveys of genomic DNA recovered from samples using our optimized AP protocol, to levels of bacteria from the phylum detected using CARD-FISH. As we did not succeed in performing a successful FISH experiment with samples from Douglas Lake, we used newly sampled and preserved samples from Muskegon Lake. We observed that 17.3% (± 2.8; 95% confidence interval) of all DAPI-stained cells hybridized to a Verrucomicrobia phylum-specific probe. This was statistically indistinguishable from the amplicon sequencing approach, in which 14.6% (± 6.0; 95% confidence interval) of all sequencing reads were annotated as Verrucomicrobia in samples recovered from the same location (extracted using the optimized AllPrep protocol). While no comparisons to the other extraction methods were made for Muskegon Lake, Verrucomicrobia levels detected in genomic DNA recovered using the optimized AllPrep method were 4–5 times higher than in DNA recovered from enzymatic and bead-beating based protocols in Douglas Lake samples.

A previous study that compared a protocol similar to the AP protocol (Qiagen DNeasy, which essentially is the DNA part of the AP protocol) to an enzymatic protocol with bead beating found an overrepresentation of amplicons from an Actinobacterium isolate in the sequencing data derived from the Qiagen kit extraction [10]. This suggests that the Qiagen lysis protocol performs well for Gram-positive bacteria. Our results showed that specific OTUs did not always follow the overall trend of the phylum (*e*.*g*., OTU2 and 76 between Douglas Lake standard and optimized AP (DL-APS vs. DL-APO-RL, S1 Table), indicating the limitations to extrapolating biases in extraction efficiencies from single representatives of a phylum to the entire phylum. The optimized AP protocol did reduce the community dissimilarity between the AP and other extraction protocols (Table 2), in part by increasing the detection levels of Actinobacteria (Fig. 2B). Optimized and standard AP extracts from Douglas Lake were run on separate sequencing runs (1 and 2; Table 1). AMOVA analysis indicated that observed community composition generated from identical DNA extracts (DL-Enz) was significantly different between these two sequencing runs (Fig. 2A, Table 2). While some OTUs that were differentially represented in the standard and optimized AP protocols were attributed to differences between the sequencing runs (S1 Table: OTU1, 5, 76), most differences between the standard and optimized protocol datasets could be ascribed to differences in the extraction protocol.

Use of RNA preservation agents significantly changed the perceived community structure relative to no treatment controls (Table 1, Fig. 2A). Our work, as well as recent studies using soil, water, and fecal samples, showed that several commercial preservation reagents bias the perceived community composition at the DNA level [36,43,44]. It is unclear why the use of these agents skews the detection levels of specific bacterial groups. In both Lake Huron and the Huron River samples the use of preservation reagents led to a decrease in the observed relative abundance of Actinobacteria, and an increase in the observed relative abundance of Bacteroidetes. This result is opposite to a previous report testing the impact of RNAlater preservation in soils [36], but is consistent with a study focused on fecal samples, where Bacteroidetes detection levels were higher in RNAlater-stored than in untreated samples [43]. Both studies used different extraction protocols than the protocols used in this study.

## Conclusion

This study contributes to the expanding body of work focused on the influence of sample preservation and extraction methods on observed bacterial community composition. The simultaneous extraction of DNA and RNA from freshwater samples collected on filters using a modified Qiagen AllPrep protocol compared favorably to other commonly used extraction protocols in terms of nucleic acid quantity and quality. Contributions to beta-diversity from differences in extraction and preservation method were smaller than from biological differences. However, since there were significant extraction method-dependent differences in the representation for specific taxa, from OTUs to phyla, our optimized method has the potential to avoid biases due to differences in extraction efficiencies when using separate single nucleic acid type extractions. The AllPrep nucleic acid extraction method outperformed other protocols we tested in representing the relative abundance of Verrucomicrobia, as assessed through *in situ* hybridization (CARD-FISH). We note that it has been suggested recently that the prevalence of this phylum in freshwater systems may have been underestimated, as several commonly used domain-level primers may poorly amplify 16S rRNA genes from these organisms [31]. To reduce technical bias in microbiome studies, we recommend (1) simultaneously extracting DNA and RNA from a single lysate (and protein and metabolites if of interest, e.g. [28]), (2) avoiding comparisons between samples preserved in protection agents and untreated samples, and (3) when possible, combining all samples from a single study on a single sequencing run.

## Materials and Methods

### Samples

No specific permissions were required for obtaining water samples from these lakes in Michigan and none of the lakes are in protected areas. The field studies did not involve endangered or protected species. Surface water samples (1–5 m below surface) originated from (a) Douglas Lake, an oligotrophic lake in Northern Michigan (Lake area = 14 km^2^; 45°33'50"N 84°40'23"W; lake depth at sampling location = 21 m; May 27, 2012; 9:05 am), (b) Lake Huron, an oligotrophic Laurentian Great Lake (59,600 km^2^; 45°0'1"N 83°22'41"W; lake depth at sampling location = 10 m; April 17, 2012, 2:05 pm), and (c) the Huron River (42°18'46"N 83°47'24"W; river depth at sampling location = 1 m; July 5, 2012 (for AP protocol optimization) and March 18, 2013, 10:30 am (for evaluation of influence of RNA preservation method)). Additional samples mentioned in the yield range originated from 108 m depth in Lake Michigan, an oligotrophic Laurentian Great Lake (58,000 km^2^; 43°11'59"N 86°34'11"W; lake depth at sampling location = 110 m; April 23, 2013, 6:30 pm), and from the surface of Muskegon Lake, a mesotrophic freshwater estuary (17 km^2^; 43°14'17"N 86°16'49"W; lake depth at sampling location = 10 m; July 16, 2013, 6:15 pm). Finally, samples used to compare CARD-FISH data to sequencing data were from the hypolimnion at three sites in Muskegon Lake, sampled between 9 am and noon on May 13, 2014. Except for the samples collected from the Muskegon Lake hypolinion, we prefiltered 10 L water for nucleic acid extraction at the sampling site using 210 and 20 μm nitex mesh. Within two hours of sampling, prefiltered water was sequentially filtered onto 142 mm 3.0 μm polycarbonate filters and 0.22 μm polyethersulfone filters (Millipore) using a Masterflex I/P peristaltic pump (Cole Parmer) between settings 11–13. The Muskegon Lake hypolimnion samples were processed similarly, but the water volume was limited to 2L and the filter diameter was 47 mm. We only used the 0.22 μm filters in this study, except for the samples from the Muskegon Lake hypolimnion, for which both 0.22–3 and 3–20 μm fractions were considered for comparison to CARD-FISH data on the 0.22–20 μm fraction. Upon water filtration in the field, filters were folded with bacterial biomass facing inwards, to fit in 50 ml falcon tubes and stored dry or submersed into 10 ml RNAlater (Ambion). The Huron River filter used to evaluate the impact of RNA preservation agents was cut into 4 equal pieces and stored either dry, in RNAlater, RNAprotect (Qiagen), or RLT+ lysis buffer (Qiagen). Samples were frozen on dry ice and transferred to a -80°C freezer. Extractions for the Huron River samples were performed after 1 day of storage, Douglas Lake and Lake Huron samples were extracted within 10 months of sampling. Samples from the Muskegon Lake hypolimnion taken for CARD-FISH (30 ml) were prefiltered (< 20 μm fraction) and fixed in 1–2% paraformaldehyde for 12–24 hrs at 4°C. We vacuum filtered fixed water samples on 0.22 μm polycarbonate filters (Millipore) and stored them at -20°C until processed for probe hybridization.

### DNA and RNA extraction

We used a combined DNA/RNA/miRNA extraction method and compared this to two commonly used DNA extraction methods and one commonly used RNA/miRNA extraction method. The latter protocol followed the Mirvana miRNA isolation kit procedure (Ambion), commonly used for filtered marine microbial communities [20,45,46].

The combined extraction method was based on the AllPrep DNA/RNA/miRNA Universal kit protocol (Qiagen). From this protocol, two types of extractions were performed which we call the standard and optimized protocols. In the standard protocol we followed the manufacturer’s manual. A segment of the 142 mm filter was cut into < 0.5 cm^2^ pieces and added to a 2 ml centrifuge tube to which the lysis buffer was added, mixed and incubated at 37°C for 1 hr. The tube was then either vortexed (at 4°C) for 10 min with or without zirconium beads (200 mg each of 0.1, 0.5 and 2 mm zirconium beads), or agitated in a Qiagen TissueLyser (2 x 1 min, 30 Hz) with the same mixture of zirconium beads. The optimized protocol used intact filter segments and added a short wash of the filter segment to remove excess RNAlater by carefully dipping the filter segment into a petri dish filled with 1X PBS (pH 7.4) and blotting the excess fluid on a nuclease-free petri dish. The optimized protocol contained a lyszoyme treatment, in which 125 μl 8 mg/ml lysozyme (Sigma) were added to the top of the filter segment, and incubated at 37°C for 5 minutes (no difference noted when compared to 30 minutes, see S2 Fig.). We inserted the filter segment into a 2 ml centrifuge tube, added lysis buffer and β-mercaptoethanol according to the manufacturer’s protocol, and incubated the mixture at room temperature for 90 minutes while rotating with a rotisserie to ensure good contact between the filter and the buffer. After this incubation, the tube was vortexed for 10 min at maximum speed without beads at 4°C, centrifuged for 15 seconds at 20,000 x g with the filter segment snapped under the cap to collect as much of the lysate as possible. The lysate was transferred to a QIAshredder column (Qiagen) and the remainder of the protocol was performed according to the manufacturer’s instructions, performing two elution steps with of 30 μl elution buffer for both RNA and DNA.

DNA extraction protocol Enz is a protocol commonly used for extracting DNA from filtered marine samples [20,34]. It uses a filter segment cut into 0.5 cm^2^ pieces and a lysis buffer composed of 40 mM EDTA, 50 mM Tris pH 8.3, 0.73 M sucrose, 1.15 mg ml^−1^ lysozyme, 200 μg ml^−1^ RNase. After 30 min incubation at 37°C while rotating with a rotisserie, a solution of 10 mg ml^−1^ Proteinase K in 40 mM EDTA, 50 mM Tris pH 8.3, and 0.73 M sucrose, as well as 1% SDS was added and incubated at 55°C for 2 hrs while rotating. Next, the DNA was extracted using a Qiagen DNeasy Tissue kit based on a modified manufacturer's protocol [34]. DNA was eluted in 200 μl elution buffer and concentrated to 40 μl using a Microcon 30 column (Millipore).

DNA extraction protocol Bead is a protocol that was developed to maximize DNA yields, used previously for marine water and sediment samples [47]. This protocol relies on combined enzymatic and harsh bead beating for cell lysis. Shortly, the filter segment cut into 0.5 cm^2^ pieces, 200 mg each of 0.1, 0.5 and 2 mm zirconium beads, and lysis solution (300 mM EDTA, 300 mM NaCl, 300 mM Tris pH 7.5, 70 μl of 15% SDS and 35 μl of 1 M DTT in 0.01 M Na acetate) were incubated at 70°C for 30 min and cooled to < 40°C. After incubating at 37°C for 20 min with 5% lysozyme (w/v in water), the mixture was agitated on a FastPrep bead beater machine for 45 s at setting 6.5. Separation of protein from nucleic acid occurs though precipitation of the SDS-protein complexes by adding 1 M KCl and centrifuging. The supernatant was concentrated using an Amicon 30 column and eluted in 100 μl of TE as described by the manufacturer (Millipore).

For each protocol, we performed triplicate extractions from segments of the same 142 mm filter. A summary of the samples used to determine the effects of different extraction methods, preservation methods, modifications to the AllPrep protocol, and sequencing run and to compare sequencing data to CARD-FISH data is presented in Table 1. Yields were measured using Picogreen and Ribogreen assays (Life Technologies), and RNA quality was evaluated based on the RNA integrity number (RIN) generated by the Agilent Bioanalyzer.

### Sequencing and analysis

DNA extracts were submitted for 16S rRNA gene amplicon sequencing performed at the University of Michigan Medical School according to Kozich *et al*. [48]. This protocol uses dual index-labeled primers that target the V4 region of bacterial and archaeal 16S rRNA genes (515F/806R) [49]. Pooled and purified libraries were sequenced on an Illumina MiSeq sequencer, using v2 chemistry 2x250 (500 cycles) paired-end reads. RTA v1.17.28 and MCS v2.2.0 software were used to generate data from four separate runs (run 1: March 12, 2013; run 2: April 28, 2013, run 3: May 31, 2013, run 4 (for CARD-FISH comparison): Jun 13, 2014). The same three replicate Enz DNA extracts, stored at -20°C since their extraction on September 9, 2012 were included in each sequencing run. We analyzed the data using mothur v.1.30.1 based on the MiSeq standard operating protocol accessed on August 13, 2014 using SILVA release 102 for alignment and classification. All statistical analysis, including the Bray-Curtis dissimilarity matrix and non-metric multidimensional scaling (NMDS) ordination generation, as well as AMOVA (10,000 iterations) and Metastats analysis were performed in mothur according to the 454 standard operating protocol [5] accessed on August 13, 2014. We rarefied the data at a subsampling level that allowed inclusion of all technical comparisons (820 subsamples) and a level that excluded only the Bead DNA extraction protocol (2,800). Sequencing data for the Muskegon Lake samples compared to CARD-FISH data were generated on a separate MiSeq sequencing run, similar to the method described above. Data analysis was performed as described above. Fastq files were submitted to NCBI sequence read archive under BioProject PRJNA271696, SRA accession number SRP051811.

### Catalyzed reporter deposition fluorescence *in situ* hybridization (CARD-FISH)

CARD-FISH was performed according to [50], with the following modifications: for the embedding step, filters were dipped into 0.1% low-gelling point agarose, placed cell-side down, and dried for 10–30 min at 35–40°C, prior to hybridization; prior to probe hybridization, filters were incubated in Image-iT Fx Signal Enhancer (Life Technologies) for 30 min at RT and then washed twice in 1x PBS; for probe hybridization, a final concentration 5 ng μl^-1^ of probes was used and incubated overnight for up to 15 hrs; for the signal amplification step, a substrate mix of Alexa Fluor 488—tyramide and amplification buffer (Life Technologies) was made based upon manufacturer’s instructions. Probes EUB338 I/II/III [51,52] were used to tag bacterial cells. Verrucomicrobia were targeted by mixing EUB338 III with unlabeled competitor probe EUB338 II to minimize non-specific hybridization. Probe NON338 was used as a negative control [53]. All probes were hybridized with 55% formamide. Filters were examined with fluorescent microscopy by taking a photo and counting the number of DAPI-stained and probe-tagged cells within the field; a minimum of 1000 DAPI-stained cells was counted per labeled filter. The reported relative abundance of bacteria belonging to the Verrucomicrobia phylum in the hypolimnion of Muskegon Lake measured by CARD-FISH is the average of three spatially separated lake samples. The 16S rRNA gene sequencing detection levels resulted from averaging data from the same three spatially separated lake samples, and combined proportional data from 0.22–3 and 3–20 μm fractions.

## Supporting Information

S1 Fig Analysis of technical bias to perceived community composition at lower subsampling level. NMDS of the 16S rRNA gene sequencing data based on a Bray-Curtis dissimilarity matrix generated after random subsampling of 820 sequences. Error bars indicate the range of coordinates for the three replicate extractions/sequencing datasets per treatment.
(TIF)

S2 Fig Lysozyme incubation time had little influence on phylum-level community composition of the three Douglas Lake samples combined into DL-APO-RL. Phylum level data (top 10 most abundant phyla, fractions of all available reads) for three variant treatments of the optimized AP protocol. As there was only one replicate per treatment, statistical testing was not possible, but since community composition was highly similar in the three treatments, we combined data as DL-APO-RL in Fig. 2. The optimized AP protocol used for other samples did include a 5 minute lysozyme treatment. Acronyms: LYS5, LYS30 = 5 and 30 minutes incubation with lysozyme, respectively; TE30 = 30 minutes incubation with TE.
(TIF)

S1 Table Identification of OTUs with significantly different representations. Taxa (OTUs) for which Metastats analysis indicated significant differences in relative abundance (p<0.05, > 1% of all sequences in at least one treatment) are listed. Metastats analysis was performed on treatments with significantly different community compositions, as indicated by AMOVA analysis.
(DOCX)
