# Supplementary figures and images for: RNA Preservation Agents and Nucleic Acid Extraction Method Bias Perceived Bacterial Community Composition
Source: PLoS One. 2015 Mar 23;10(3):e0121659. doi: 10.1371/journal.pone.0121659 (PMC4370824; doi:10.1371/journal.pone.0121659)

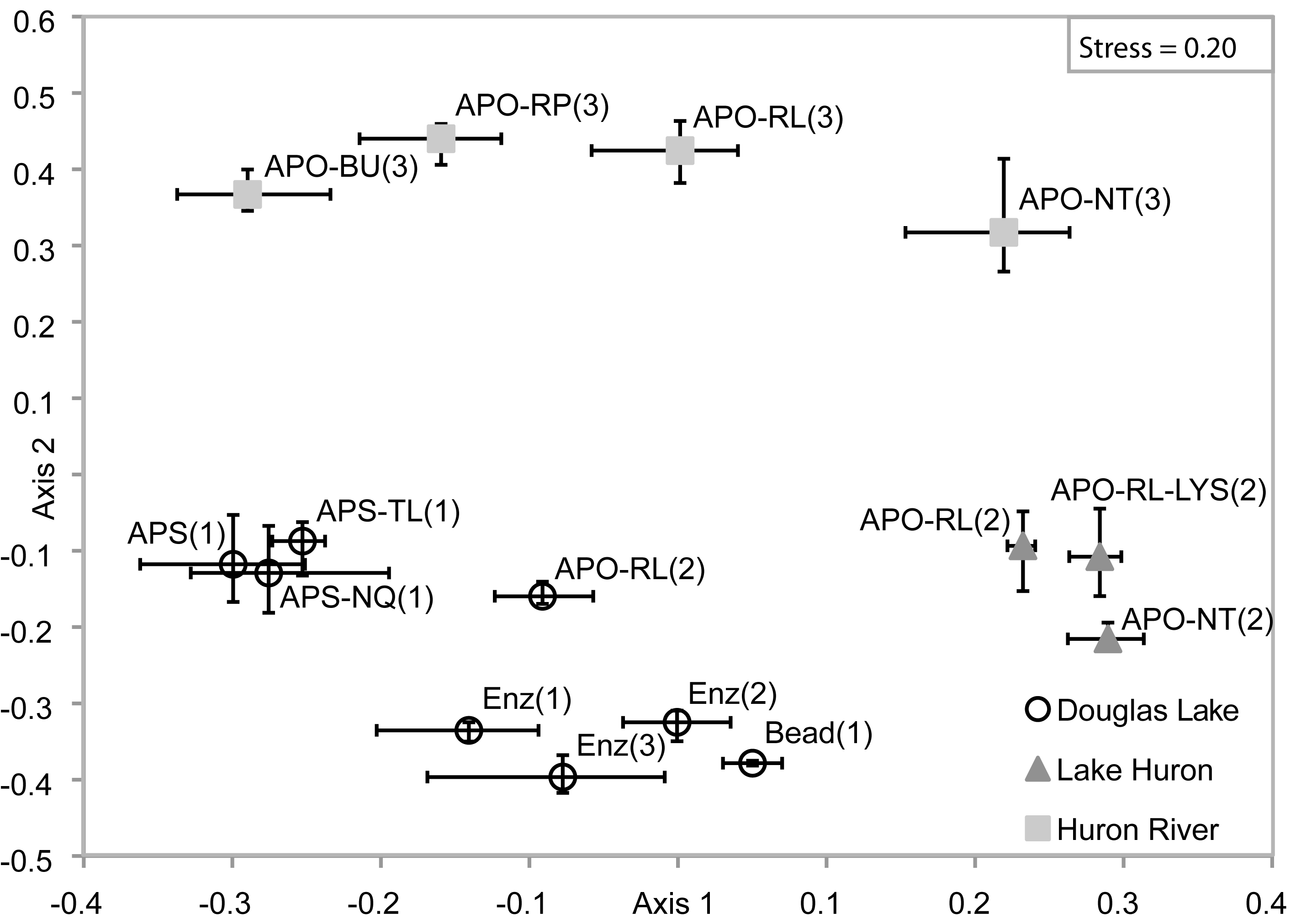

Supplement: S1 Fig — NMDS of the 16S rRNA gene sequencing data based on a Bray-Curtis dissimilarity matrix generated after random subsampling of 820 sequences. Error bars indicate the range of coordinates for the three replicate extractions/sequencing datasets per treatment. (TIF) [file pone.0121659.s001.tif]

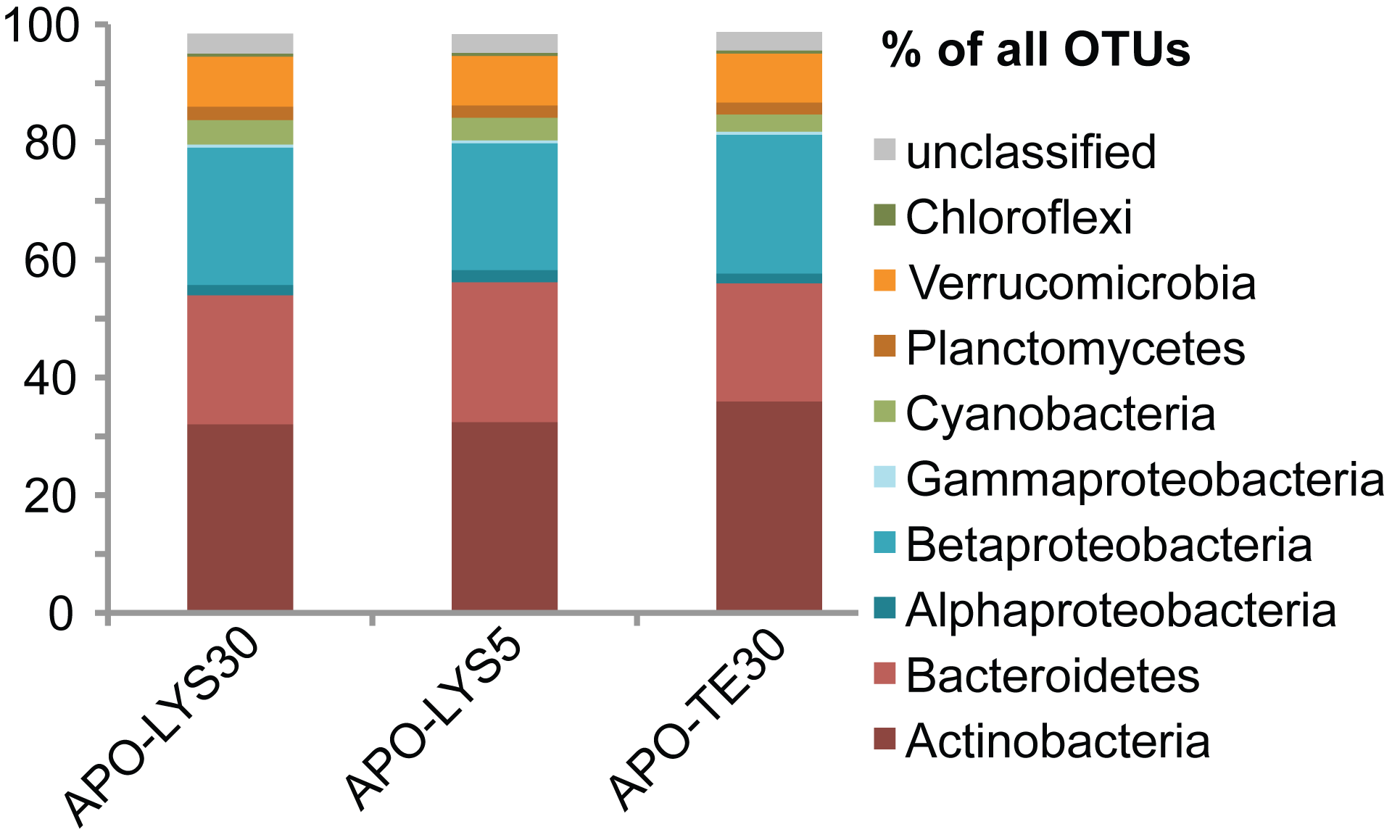

Supplement: S2 Fig — Phylum level data (top 10 most abundant phyla, fractions of all available reads) for three variant treatments of the optimized AP protocol. As there was only one replicate per treatment, statistical testing was not possible, but since community composition was highly similar in the three treatments, we combined data as DL-APO-RL in Fig. 2. The optimized AP protocol used for other samples did include a 5 minute lysozyme treatment. Acronyms: LYS5, LYS30 = 5 and 30 minutes incubation with lysozyme, respectively; TE30 = 30 minutes incubation with TE. (TIF) [file pone.0121659.s002.tif]
