# Supplementary material for: RNA Preservation Agents and Nucleic Acid Extraction Method Bias Perceived Bacterial Community Composition
Source: PLoS One. 2015 Mar 23;10(3):e0121659. doi: 10.1371/journal.pone.0121659 (PMC4370824; doi:10.1371/journal.pone.0121659)
Supplement: S1 Table — Taxa (OTUs) for which Metastats analysis indicated significant differences in relative abundance (p<0.05, > 1% of all sequences in at least one treatment) are listed. Metastats analysis was performed on treatments with significantly different community compositions, as indicated by AMOVA analysis. (DOCX) [file pone.0121659.s003.docx]

**Supplemental Table.**

**Table S1: Identification of OTUs with significantly different representations.** We listed OTUs for which Metastats analysis indicated significant differences in relative abundance (p<0.05, > 1% of all sequences in at least one treatment). Data is presented only for comparisons between extraction methods, preservation methods, or sequencing runs, which AMOVA showed a statistically significant difference between.

| **OTU** | **DL-Enz** | **DL-APO-RL** | **p-value** | **Taxonomy** |
| --- | --- | --- | --- | --- |
| Otu00005 | 0.081 | 0.061 | 0.046 | Proteobacteria_Betaproteobacteria_Burkholderiales_Burkholderiaceae_Polynucleobacter |
| Otu00006 | 0.021 | 0.030 | 0.040 | Bacteroidetes_Sphingobacteria_Sphingobacteriales_Chitinophagaceae_Sediminibacterium |
| Otu00007 | 0.017 | 0.044 | 0.000 | Bacteroidetes_Flavobacteria_Flavobacteriales_Cryomorphaceae_Fluviicola |
| Otu00016 | 0.015 | 0.018 | 0.049 | Bacteroidetes_Flavobacteria_Flavobacteriales_Flavobacteriaceae_Flavobacterium |
| Otu00017 | 0.005 | 0.010 | 0.003 | Bacteroidetes_Flavobacteria_Flavobacteriales_Flavobacteriaceae_Flavobacterium |
| Otu00019 | 0.001 | 0.016 | 0.002 | Cyanobacteria |
| Otu00020 | 0.029 | 0.045 | 0.012 | Proteobacteria_Betaproteobacteria_Methylophilales_Methylophilaceae_LD28 |
| Otu00026 | 0.011 | 0.024 | 0.003 | Bacteroidetes_Sphingobacteria_Sphingobacteriales_Cyclobacteriaceae_uncultured |
| Otu00043 | 0.002 | 0.015 | 0.000 | Bacteroidetes_Sphingobacteria_Sphingobacteriales |
| Otu00055 | 0.003 | 0.024 | 0.002 | Verrucomicrobia |
| Otu00057 | 0.002 | 0.028 | 0.000 | Verrucomicrobia_Acidimethylosilex |
| Otu00059 | 0.086 | 0.008 | 0.008 | Planctomycetes_Planctomycetacia_Planctomycetales_Planctomycetaceae |
| Otu00066 | 0.016 | 0.009 | 0.001 | Actinobacteria_Actinobacteria_Actinobacteridae_Actinomycetales |
| Otu00068 | 0.059 | 0.021 | 0.011 | Cyanobacteria_SubsectionI_Synechococcus |
| Otu00079 | 0.006 | 0.012 | 0.000 | Bacteroidetes |
|  |  |  |  |  |
| **OTU** | **DL-Enz** | **DL-APS** | **p-value** |  |
| Otu00001 | 0.102 | 0.074 | 0.008 | Proteobacteria_Betaproteobacteria_Burkholderiales_Comamonadaceae_Rhodoferax |
| Otu00002 | 0.120 | 0.082 | 0.001 | Actinobacteria_Actinobacteria_Actinobacteridae_Actinomycetales_Frankineae_Sporichthyaceae_hgcI_clade |
| Otu00004 | 0.097 | 0.066 | 0.009 | Actinobacteria_Actinobacteria_Actinobacteridae_Actinomycetales_Frankineae_Sporichthyaceae_hgcI_clade |
| Otu00005 | 0.081 | 0.031 | 0.001 | Proteobacteria_Betaproteobacteria_Burkholderiales_Burkholderiaceae_Polynucleobacter |
| Otu00007 | 0.017 | 0.042 | 0.001 | Bacteroidetes_Flavobacteria_Flavobacteriales_Cryomorphaceae_Fluviicola |
| Otu00019 | 0.001 | 0.026 | 0.001 | Cyanobacteria |
| Otu00021 | 0.011 | 0.008 | 0.006 | Proteobacteria_Betaproteobacteria_Burkholderiales_Comamonadaceae |
| Otu00022 | 0.001 | 0.013 | 0.001 | Planctomycetes_Phycisphaerae_Phycisphaerales_Phycisphaeraceae_CL500-3 |
| Otu00026 | 0.011 | 0.027 | 0.001 | Bacteroidetes_Sphingobacteria_Sphingobacteriales_Cyclobacteriaceae_uncultured |
| Otu00028 | 0.004 | 0.010 | 0.001 | Bacteroidetes_Flavobacteria_Flavobacteriales_Flavobacteriaceae_Flavobacterium |
| Otu00041 | 0.006 | 0.014 | 0.001 | Bacteroidetes_Sphingobacteria_Sphingobacteriales_Chitinophagaceae_uncultured |
| Otu00043 | 0.002 | 0.018 | 0.001 | Bacteroidetes_Sphingobacteria_Sphingobacteriales |
| Otu00049 | 0.012 | 0.008 | 0.005 | Actinobacteria_Actinobacteria_Actinobacteridae_Actinomycetales_Frankineae_Sporichthyaceae_hgcI_clade |
| Otu00055 | 0.003 | 0.049 | 0.001 | Verrucomicrobia |
| Otu00059 | 0.086 | 0.004 | 0.001 | Planctomycetes_Planctomycetacia_Planctomycetales_Planctomycetaceae |
| Otu00066 | 0.016 | 0.005 | 0.001 | Actinobacteria_Actinobacteria_Actinobacteridae_Actinomycetales |
| Otu00073 | 0.001 | 0.011 | 0.001 | Verrucomicrobia_Opitutae_vadinHA64 |
| Otu00074 | 0.015 | 0.011 | 0.044 | Actinobacteria_Actinobacteria_Actinobacteridae_Actinomycetales_Frankineae_Sporichthyaceae_hgcI_clade |
| Otu00079 | 0.006 | 0.015 | 0.001 | Bacteroidetes |
| Otu00094 | 0.003 | 0.016 | 0.001 | Verrucomicrobia_Verrucomicrobiae_Verrucomicrobiales_Verrucomicrobiaceae |
| Otu00115 | 0.003 | 0.010 | 0.001 | Bacteroidetes_Sphingobacteria_Sphingobacteriales_Saprospiraceae |
| Otu00126 | 0.007 | 0.016 | 0.002 | Verrucomicrobia_Verrucomicrobiae_Verrucomicrobiales_Verrucomicrobiaceae_Prosthecobacter |
|  |  |  |  |  |
| **OTU** | **DL-APS** | **DL-APO-RL** | **p-value** |  |
| Otu00001 | 0.074 | 0.099 | 0.005 | Proteobacteria_Betaproteobacteria_Burkholderiales_Comamonadaceae_Rhodoferax |
| Otu00002 | 0.082 | 0.103 | 0.038 | Actinobacteria_Actinobacteria_Actinobacteridae_Actinomycetales_Frankineae_Sporichthyaceae_hgcI_clade |
| Otu00005 | 0.031 | 0.061 | 0.002 | Proteobacteria_Betaproteobacteria_Burkholderiales_Burkholderiaceae_Polynucleobacter |
| Otu00022 | 0.013 | 0.008 | 0.021 | Planctomycetes_Phycisphaerae_Phycisphaerales_Phycisphaeraceae_CL500-3 |
| Otu00028 | 0.010 | 0.007 | 0.014 | Bacteroidetes_Flavobacteria_Flavobacteriales_Flavobacteriaceae_Flavobacterium |
| Otu00030 | 0.003 | 0.012 | 0.000 | unclassified |
| Otu00032 | 0.021 | 0.010 | 0.001 | Candidate_division_OP10 |
| Otu00041 | 0.014 | 0.004 | 0.001 | Bacteroidetes_Sphingobacteria_Sphingobacteriales_Chitinophagaceae_uncultured |
| Otu00049 | 0.008 | 0.014 | 0.001 | Actinobacteria_Actinobacteria_Actinobacteridae_Actinomycetales_Frankineae_Sporichthyaceae_hgcI_clade |
| Otu00055 | 0.049 | 0.024 | 0.025 | Verrucomicrobia |
| Otu00057 | 0.005 | 0.028 | 0.000 | Verrucomicrobia_Acidimethylosilex |
| Otu00068 | 0.044 | 0.021 | 0.004 | Cyanobacteria_SubsectionI_Synechococcus |
| Otu00076 | 0.018 | 0.009 | 0.040 | Actinobacteria_Actinobacteria_Acidimicrobidae_Acidimicrobiales_Acidimicrobineae_Acidimicrobiaceae_marine_group |
| Otu00079 | 0.015 | 0.012 | 0.021 | Bacteroidetes |
| Otu00094 | 0.016 | 0.007 | 0.001 | Verrucomicrobia_Verrucomicrobiae_Verrucomicrobiales_Verrucomicrobiaceae |
| Otu00115 | 0.010 | 0.003 | 0.000 | Bacteroidetes_Sphingobacteria_Sphingobacteriales_Saprospiraceae |
| Otu00126 | 0.016 | 0.006 | 0.004 | Verrucomicrobia_Verrucomicrobiae_Verrucomicrobiales_Verrucomicrobiaceae_Prosthecobacter |
|  |  |  |  |  |
| **OTU** | **HR-APO-RP** | **HR-APO-NT** | **p-value** |  |
| Otu00001 | 0.085 | 0.143 | 0.043 | Proteobacteria_Betaproteobacteria_Burkholderiales_Comamonadaceae_Rhodoferax |
| Otu00004 | 0.010 | 0.024 | 0.018 | Actinobacteria_Actinobacteria_Actinobacteridae_Actinomycetales_Frankineae_Sporichthyaceae_hgcI_clade |
| Otu00006 | 0.010 | 0.018 | 0.038 | Bacteroidetes_Sphingobacteria_Sphingobacteriales_Chitinophagaceae_Sediminibacterium |
| Otu00011 | 0.010 | 0.022 | 0.007 | Proteobacteria_Betaproteobacteria_Methylophilales_Methylophilaceae_Methylophilus |
| Otu00019 | 0.060 | 0.004 | 0.006 | Cyanobacteria |
| Otu00069 | 0.013 | 0.007 | 0.034 | Bacteroidetes |
| Otu00164 | 0.005 | 0.012 | 0.007 | Bacteroidetes_Sphingobacteria_Sphingobacteriales_env.OPS_17 |
|  |  |  |  |  |
| **OTU** | **HR-APO-NT** | **HR-APO-RL** | **p-value** |  |
| Otu00004 | 0.024 | 0.011 | 0.030 | Actinobacteria_Actinobacteria_Actinobacteridae_Actinomycetales_Frankineae_Sporichthyaceae_hgcI_clade |
| Otu00007 | 0.008 | 0.014 | 0.037 | Bacteroidetes_Flavobacteria_Flavobacteriales_Cryomorphaceae_Fluviicola |
| Otu00019 | 0.004 | 0.029 | 0.005 | Cyanobacteria |
| Otu00043 | 0.005 | 0.011 | 0.013 | Bacteroidetes_Sphingobacteria_Sphingobacteriales |
| Otu00136 | 0.007 | 0.012 | 0.040 | Planctomycetes_Planctomycetacia_Planctomycetales_Planctomycetaceae |
| Otu00164 | 0.012 | 0.007 | 0.014 | Bacteroidetes_Sphingobacteria_Sphingobacteriales_env.OPS_17 |
|  |  |  |  |  |
| **OTU** | **HR-APO-NT** | **HR-APO-BU** | **p-value** |  |
| Otu00001 | 0.143 | 0.066 | 0.031 | Proteobacteria_Betaproteobacteria_Burkholderiales_Comamonadaceae_Rhodoferax |
| Otu00006 | 0.018 | 0.010 | 0.039 | Bacteroidetes_Sphingobacteria_Sphingobacteriales_Chitinophagaceae_Sediminibacterium |
| Otu00014 | 0.026 | 0.015 | 0.009 | Bacteroidetes_Sphingobacteria_Sphingobacteriales_Cytophagaceae_Arcicella |
| Otu00019 | 0.004 | 0.094 | 0.005 | Cyanobacteria |
| Otu00136 | 0.007 | 0.019 | 0.015 | Planctomycetes_Planctomycetacia_Planctomycetales_Planctomycetaceae |
| Otu00164 | 0.012 | 0.003 | 0.006 | Bacteroidetes_Sphingobacteria_Sphingobacteriales_env.OPS_17 |
| Otu00184 | 0.006 | 0.017 | 0.014 | Planctomycetes_Planctomycetacia_Planctomycetales_Planctomycetaceae_uncultured |
|  |  |  |  |  |
| **OTU** | **LH-APO-NT** | **LH-APO-RL/RL-LYS** | **p-value** |  |
| Otu00003 | 0.015 | 0.022 | 0.035 | Bacteroidetes_Flavobacteria_Flavobacteriales_Flavobacteriaceae_Flavobacterium |
| Otu00004 | 0.184 | 0.118 | 0.001 | Actinobacteria_Actinobacteria_Actinobacteridae_Actinomycetales_Frankineae_Sporichthyaceae_hgcI_clade |
| Otu00008 | 0.062 | 0.054 | 0.035 | Actinobacteria_Actinobacteria_Actinobacteridae_Actinomycetales_Frankineae_Sporichthyaceae_hgcI_clade |
| Otu00009 | 0.003 | 0.014 | 0.003 | Verrucomicrobia_Opitutae_vadinHA64 |
| Otu00012 | 0.017 | 0.024 | 0.038 | Bacteroidetes_Flavobacteria_Flavobacteriales_Flavobacteriaceae_Flavobacterium |
| Otu00020 | 0.026 | 0.041 | 0.002 | Proteobacteria_Betaproteobacteria_Methylophilales_Methylophilaceae_LD28 |
| Otu00049 | 0.019 | 0.013 | 0.004 | Actinobacteria_Actinobacteria_Actinobacteridae_Actinomycetales_Frankineae_Sporichthyaceae_hgcI_clade |
|  |  |  |  |  |
| **OTU** | **DL-Enz(2)** | **DL-Enz(1)** | **p-value** |  |
| Otu00001 | 0.125 | 0.069 | 0.002 | Proteobacteria_Betaproteobacteria_Burkholderiales_Comamonadaceae_Rhodoferax |
| Otu00005 | 0.086 | 0.064 | 0.016 | Proteobacteria_Betaproteobacteria_Burkholderiales_Burkholderiaceae_Polynucleobacter |
| Otu00020 | 0.029 | 0.022 | 0.010 | Proteobacteria_Betaproteobacteria_Methylophilales_Methylophilaceae_LD28 |
| Otu00060 | 0.004 | 0.014 | 0.004 | Actinobacteria_Actinobacteria_Acidimicrobidae_Acidimicrobiales_Acidimicrobineae_Acidimicrobiaceae |
| Otu00076 | 0.007 | 0.025 | 0.006 | Actinobacteria_Actinobacteria_Acidimicrobidae_Acidimicrobiales_Acidimicrobineae_Acidimicrobiaceae_marine_group_marine_group |
| Otu00132 | 0.005 | 0.014 | 0.003 | Actinobacteria_Actinobacteria_Acidimicrobidae_Acidimicrobiales_Acidimicrobineae |
| Otu00136 | 0.005 | 0.011 | 0.036 | Planctomycetes_Planctomycetacia_Planctomycetales_Planctomycetaceae |
| Otu00227 | 0.006 | 0.013 | 0.010 | Actinobacteria_Actinobacteria_Actinobacteridae_Actinomycetales |
